# Editorial: Molecular mechanisms of proteinuria, volume II

**DOI:** 10.3389/fmed.2022.1026202

**Published:** 2022-10-13

**Authors:** Ilse S. Daehn, Sandra Merscher

**Affiliations:** ^1^Division of Nephrology, Department of Medicine, The Icahn School of Medicine at Mount Sinai, New York, NY, United States; ^2^Katz Family Division of Nephrology and Hypertension, Department of Medicine, Peggy and Harold Katz Family Drug Discovery Center, University of Miami, Miami, FL, United States

**Keywords:** proteinuria, glomerulus, kidney disease, mechanisms of disease, models of disease

Proteinuria, which is defined as persistent and increased leakage of protein in the urine, is a consequence of an altered glomerular filtration and is considered a key marker for renal barrier dysfunction. In support, the recent recognition of the predictive link between proteinuria and disease progression to organ failure in kidney diseases has led to recommend proteinuria as an approvable primary endpoint by regulatory organizations, which will allow for the increased evaluation of investigational therapies in patients with kidney disease and accelerate the drug discovery process (1, 2). However, the molecular mechanisms leading to the development of proteinuria are complex, and there is a need to decipher these for a new generation of therapeutic drugs to treat the millions of people suffering from kidney disease.

This Research Topic represents a collection of several research articles, mini-reviews and reviews by our esteemed colleagues that are leaders in the field and put forth the current state-of-the-art of some of the key pathological mechanisms underlying proteinuric kidney diseases. The manuscripts in this Topic present exciting technologies aimed to uncover targets with therapeutic potential, as well as the glomerular cell specific contributions in the maintenance or in the breakdown of the glomerular filtration barrier. We are also thrilled that there is a diverse representation including female and minority researchers in STEM as first and senior corresponding authors participating in this Research Topic. We hope that this is a trend toward a more diverse and inclusive representation of emerging leaders in field.

In several original research articles of this collection, authors describe their discoveries of novel mechanisms that contribute to podocyte injury and development of proteinuria. Basgen et al. demonstrate that podocyte injury is the initiating event leading to albuminuria and mesangial expansion in the *Cd2ap* KO mouse model of focal segmental glomerulosclerosis (FSGS). The authors highlight that the sequence of events, starting with podocyte injury, has important implications, particularly in underlying proteinuric disease pathology. Li et al. describe a crucial role for microRNA-146a (miR-146a) in podocyte injury in non-diabetic and diabetic kidney disease. They demonstrate that mice that lack podocyte miR-146a develop proteinuria and glomerular injury and confirmed its role in protecting podocytes in diabetic kidney disease (DKD) (Li et al.). Similarly, research by Song et al. unveils an essential role for podocyte MCC regulator of WNT signaling pathway (MCC), whereby reduced expression of MCC, which can be observed in glomeruli of diabetic mice and patients with FSGS, results in the loss of lamellipodia *in vitro*. Lastly, Azzam et al. investigated the interplay between reactive oxygen species (ROS) and sphingomyelin phosphodiesterase acid like 3b (SMPDL3B) in mediating the response to radiation-induced injury of podocytes and identify NOX-derived reactive oxygen species (ROS) as a novel upstream regulator of SMPDL3b.

Indeed, podocytes are central in the pathogenesis of proteinuria, and this collection also offers reviews and mini-reviews focusing on the role of other glomerular cells as critical players in the pathogenesis of glomerular injury and proteinuria. First, the role of the mesangial cells in glomerular function and intra-glomerular crosstalk is discussed in depth in a review by Ebefors et al. The authors highlight current literature that supports the active involvement of the mesangium in disease onset and progression. Next, the role of glomerular endothelial cells in proteinuria is reviewed by Ballermann et al. The review summarizes the vast literature that supports the importance of this monolayer in blocking large plasma proteins from passing into the glomerular filtrate and explains how the interaction of the glycocalyx with its endothelial surface layer contributes to the maintenance of the barrier function (Ballermann et al.). Further, the authors discuss the impact of glomerular endothelial cell dysfunction, de-differentiation and activation of endothelial cells in various diseases. The review by Gyarmati et al. centers on the role of endothelial cell injury and microthrombi in podocyte detachment and albumin leakage *via* hemodynamic and mechanical forces. The authors discuss how alterations of the glycocalyx and its interaction with the microenvironment represents a key pathogenic mechanism that results in proteinuria in various diseases (Gyarmati et al.), adding to our growing understanding of the glomerular cell specific roles in the pathogenesis of proteinuria.

Abnormalities of glomerular cell function due to mitochondrial defects is involved in the etiology of proteinuric glomerular diseases (3). In the review by Galvan et al., the authors discuss the latest discoveries that advance our understanding of the nature of mitochondrial dysfunction and how it could contribute to the progression of DKD. The authors highlight specific knowledge gaps as well as the potential for new therapeutics targeting mitochondrial dysfunction in DKD.

The mini-review by Wei et al. summarizes the role of urokinase-type plasminogen activator receptor (uPAR) in podocyte injury and in proteinuric kidney disease. The authors discuss the evidence supporting a role for elevated serum suPAR as a circulating risk factor for kidney diseases and how the complexity of suPAR derived from different enzymatic cleavage and other modifications may have confounded the determination of serum suPAR levels in patients in previous studies. In a review by Bisgaard and Christoffersen, the role of kidney-derived and plasma APOM is discussed in the context of kidney disease development. The authors highlight recent research suggesting that changes in the APOM/S1P axis contribute to the pathogenesis of kidney diseases and could be a potential therapeutic target (Bisgaard and Christoffersen). The molecular mechanisms of proteinuria in Minimal Change Disease (MCD) are reviewed by Purohit et al. and shed light on the complex interplay between the immune system, glomerular cells, and the genome, raising the possibility of distinct underlying mechanisms of proteinuria among patients with MCD. Hall and Wyatt review the mechanisms of proteinuria in HIV-associated nephropathy (HIVAN). The roles of injury induced podocyte dedifferentiation, hyperplasia, cytoskeletal dysregulation and apoptosis in HIVAN, and the role of apolipoprotein L1 (APOL1) risk variants are discussed. Indeed insights from HIVAN could help improve the management of COVID-19-associated kidney disease given the similar clinical presentations, pathological findings and potential disease mechanisms (4).

New and emerging technologies to study the molecular mechanisms of proteinuria are also presented in this Topic. The mini review by Latt et al. summarizes how single-cell and single-nucleus RNA sequencing approaches have improved our understanding of the pathophysiology of glomerular diseases at a cellular level. They argue that these methods could be useful in exploring non-invasive approaches to aid in the identification of precision therapeutics for proteinuric diseases (Latt et al.). Gong et al. discuss the opportunities for single cell RNA sequencing and bioinformatics-based spatial transcriptomics. In this context, the authors review how other novel approaches such as spatial transcriptomics, the development of the glomerulus-on-a-chip and of kidney organoids are contributing to our growing understanding of glomerular pathophysiology (Gong et al.). Advances in genetics have led to the remarkable discovery that the presence of two high-risk variants of Apolipoprotein L1 (APOL1) in people of recent African ancestry confers susceptibility to the development of several glomerular diseases, including FSGS and HIVAN. The mini review by Yoshida et al. discusses the advantages and limitations of several animal models, including mice, zebrafish and drosophila that have been used to study APOL1 function in health and disease. Forward thinking research will leverage these endeavors to unlock mechanistic insights that will open new avenues for discoveries in this space.

It is an exciting time in our field as in the past few years we have witnessed the development of new technologies and improved methods, that have led to the discovery of novel targets within the glomerular filtration barrier and application of new drugs currently undergoing clinical evaluation (5). The number of compounds in all phases of clinical trials for kidney diseases has doubled in the past decade and we anticipate that the number will continue to increase in the next few years.

## Author contributions

All authors listed have made a substantial, direct, and intellectual contribution to the work and approved it for publication.

## Funding

ID was supported by the National Institutes of Health grant R01DK097253 and Department of Defense CDMRP grants W81XWH-20-1-0836. SM was supported by the NIH grants R01DK117599, R01DK104753, and R01CA227493 by the Katz Family Division of Nephrology and Hypertension and the Peggy and Harold Katz Family Drug Discovery Center of the University of Miami, Miami, FL, and by Aurinia Pharmaceuticals.

## Conflict of interest

SM is an inventor on pending or issued patents (PCT/US11/56272, PCT/US12/62594, PCT/US2019/041730, PCT/US2019/032215, PCT/US13/36484, and PCT 62/674,897) aimed at diagnosing or treating proteinuric kidney diseases and stands to gain royalties from the future commercialization of these patents. SM holds equity interest in L&F Research who has licensed worldwide rights to develop and commercialize hydroxypropyl-beta-cyclodextrin to ZyVersa Therapeutics, Inc. The remaining author declares that the research was conducted in the absence of any commercial or financial relationships that could be construed as a potential conflict of interest.

## Publisher's note

All claims expressed in this article are solely those of the authors and do not necessarily represent those of their affiliated organizations, or those of the publisher, the editors and the reviewers. Any product that may be evaluated in this article, or claim that may be made by its manufacturer, is not guaranteed or endorsed by the publisher.
